# 

**Published:** 1887-02-05

**Authors:** 


PRICE ONE PENNY.

No. 19, Vol. I.

February 5th, 1887.

With a Special Supplement of Eight Pages.

Per Annum, 6s. 6d., post free.
Monthly Parts, 6d.; post free, 7½d.
Ditto per Ann., 7s. 6d., post free.

